# Mannitol and the blood-labyrinth barrier

**DOI:** 10.1186/s40463-017-0245-8

**Published:** 2017-12-11

**Authors:** Trung N. Le, Brian W. Blakley

**Affiliations:** 1 Department of Otolaryngology – Head & Neck Surgery, Sunnybrook Research Institute, University of Toronto, 2075 Bay view Ave., Room M1.102, Toronto, ON M4N 3M5 Canada; 2 Department of Otolaryngology – Head & Neck Surgery, University of Manitoba, GB421 - 820 Sherbrook Street, Winnipeg, MB R3A 1R9 Canada

**Keywords:** Blood labyrinth barrier, Blood brain barrier, Cerebrospinal fluid, Perilymph, Gentamicin, Mannitol, Pharmacokinetics, Permeability

## Abstract

**Background:**

Characterization of the blood labyrinth barrier (BLB) is extremely important to determine whether the BLB can be manipulated pharmacologically. However, experiments to investigate the BLB are technically difficult to perform. In this report, we demonstrated a unique method of controlling the BLB, and established the pharmacokinetics of gentamicin in perilymph, cerebrospinal fluid (CSF) and blood with and without mannitol.

**Study design:**

Controlled animal research project.

**Methods:**

Permeability of the BLB and the blood brain barrier (BBB) to gentamicin with and without mannitol was studied by collecting 175 samples from 44 guinea pigs using concentrations relevant to human clinical situations. *Samples were taken from two groups of 22 animals, with each animal undergoing sampling at a different time after administration of either 10 mg/ml gentamicin (4 mg/kg) (Gardena, CA) alone or gentamicin with 20% mannitol (250 mg/kg) (Mallinckrodt Inc., KY). The sample times varied from 0.5 to 17.5 h post-infusion. Samples were also taken from 4 animals as negative controls after administration of normal saline. Our goal was to simultaneously assess the pharmacokinetics of gentamicin in each of three different fluid samples in the same animal. Thus at the pre-determined post-infusion sampling time, each animal was sampled once for perilymph, CSF, and blood before being euthanized. Each animal contributed to a single time point on the subsequent pharmacokinetic curves with more than one animal per time point.*

**Results:**

Mannitol increased the rate of entry and egress of gentamicin through BLB significantly (*p* = 0.0044) but the effects on the BBB did not reach statistical significance (*p* = 0.581). Mannitol did not alter renal clearance of gentamicin from the blood (*p* = 0.433). The concentration of gentamicin in perilymph and CSF was always significantly lower than in blood.

**Conclusions:**

Mannitol administration transiently increases the permeability of the BLB. Potential clinical benefits may accrue from selected timing of administration of osmotic agents such as mannitol augmenting the rate of entry and egress of compounds such as gentamicin into and out of perilymph.

## Background

The existence of blood-brain, blood-cerebrospinal fluid, and blood-aqueous barriers have been known for many years [1, 2]. The blood labyrinth barrier (BLB) is a physiological barrier that prevents some compounds, particularly of high molecular weight, from crossing from the blood into the perilymph of the inner ear [3, 4]. The BLB is thought to have similarities to the blood-brain barrier (BBB) which has been explored much more widely [5]. Mannitol can transiently alter the permeability of the BBB [6]. This phenomenon is under study as a possible way to allow drugs to enter the cerebrospinal fluid (CSF) [7].

Mannitol is an osmotic agent. Osmotic agents such as glycerol can affect hearing and have been used diagnostically for Meniere’s disease [8]. Mannitol acts as a radical scavenger and iron chelator to attenuate gentamicin ototoxicity in guinea pig & rat in vivo [9–11]*.* The antibacterial efficacy of aminoglycosides remains uncompromised by co-therapy with mannitol in guinea pig in vivo [12]; however, there is a lack of quantitative knowledge of the therapeutic use of mannitol in the BLB.

In an unpublished pilot study we found that mannitol injection increased perilymph osmolality higher than serum, similar to the results of others [2]. The goal of the current study is to apply accepted pharmacokinetic techniques to quantify the entry and clearance of gentamicin in perilymph, CSF, and blood with and without mannitol. If mannitol changes the permeability of the BLB it is likely that this information can be applied therapeutically.

If these results are to be applied therapeutically in humans, we believe that the drugs must be delivered to test animals in doses that approximate those that might be given to humans. Most of the in vivo research on gentamicin toxicity in animals utilizes doses of gentamicin that exceed toxic human doses by several orders of magnitude [13–15]. These massive doses can potentially introduce artifacts and overwhelm different trafficking routes such as tight junctions, stria vascularis, modiolus, basilar membrane, spiral ligament [16]. Our study used clinically relevant doses of gentamicin and mannitol that applied to common human treatments and still allowed for measurement and calculation of their phamacokinetics.

## Methods

The guinea pig was chosen because its hearing and vestibular systems are very similar to those of humans, as well as its ease of handling and large size of the cochlea [17]. A total of 175 samples of perilymph, blood and CSF were collected from 44 Dunkin-Hartley guinea pigs (Charles River Breeding Lab, Senneville) with jugular vein catheters placed for intravenous injection. Samples were taken from two groups of 22 animals, each at different times after administration of either 10 mg/ml gentamicin (4 mg/kg) (Gardena, CA) alone or gentamicin with 20% mannitol (250 mg/kg) (Mallinckrodt Inc., KY). Samples were also taken from 4 animals as negative controls after administration of normal saline. Our goal was to simultaneously assess the pharmacokinetics of gentamicin in each of three different fluid samples. Each animal was sampled once for perilymph, CSF, and blood before it was terminally collected at each individual post-infusion time varying from 0.5 to 17.5 h. Each animal contributed to a single time point on the subsequent pharmacokinetic curves with more than one animal per time point.

All infusions were delivered via cannula inserted into the left external jugular vein with an infusion pump at a constant infusion rate of 0.3 ml/min. The protocol was approved by the University of Manitoba Animal Research Ethics Committee.

Prior to this project, a pilot project was undertaken that helped identify the methods, feasibility and time required to collect samples of all three fluids at similar times. We recorded the exact times of sampling after administration. Perlymph, CSF, and blood samples in the same animal were collected within 15–20 min of each other.

### Sampling procedures

Perilymph sampling was carried by surgically identifying the round window under general anesthetic with isoflurane using an operating microscope. Then the round window was pierced and a capillary tube (Drummond Scientific, PA) was inserted into the scala tympani. A maximum of 4–6 μl of perilymph fluid was successfully obtained from a cochlea. Micropipettes were sealed with wax and stored at 4 °C and analyzed within 24 h.

CSF sampling was performed by incising the skin and soft tissue over the occipital bone, carrying the dissection down to the atlanto-occipital ligament which was exposed and incised, entering the cisterna magna. This created free flow of CSF. A micropipette was inserted into the CSF pool obtaining 3–8 μl of fluid.

Blood was obtained by cardiac aspiration under the same terminal general anesthetic as the other samples. After allowing the blood to clot and centrifuging the sample, a micropipette was used to collect 4–8 μl of serum.

Some perilymph and CSF samples were contaminated with blood as evident during surgery and sample collection and not analyzed. In the 44 animals (88 ears) in the gentamicin and gentamicin with mannitol groups, five perilymph samples in the gentamicin group and 4 in the gentamicin with mannitol group were excluded for this reason. Four CSF samples in the gentamicin group and 3 in the gentamicin with mannitol group were excluded because they were contaminated with blood. The remaining samples were adequate for convergence of the parameter estimates for function fitting by GRAHPAD PRISM5 software.

### Gentamicin assay

Enzyme-linked Immunoassay (ELISA) Test Kits (Bioo Scientific, TX) were used to measure gentamicin levels in perilymph, CSF, and blood. This assay has adequate sensitivity for concentrations of gentamicin that are an order of magnitude less than those encountered in this study. In summary, 2 μl of each sample was diluted 80× using sample extraction buffer. Triplicates of 50 μl of each diluted sample were subject to the competitive enzyme immunoassay in the ELISA plate for the quantitative analysis of gentamicin as per the manufacturer’s instructions protocol. The ELISA plate was read using Gemini XPS microplate reader (Molecular Devices, CA) with 450 nm wavelength. The gentamicin concentrations (ng/ml) of the tested samples were determined from a 6-points standard curve with a negative control. A total of 175 data points were collected from control and tested animals (gentamicin with or without mannitol) in addition to 6 samples “spiked” with known concentrations of gentamicin.

### Statistical analysis

After verifying the validity of testing with the “spiked” samples and subtracting the non-specific background levels estimated from the saline-treated controls so that the expected pre-treatment and final asymptotic gentamicin concentrations equal zero, the gentamicin levels were entered into a database and analyzed using GRAPH PAD PRISM 5.0 software. The software provided best-fit parameter estimates with 95% confidence interval estimations and compared data fit between gentamicin with mannitol and gentamicin without mannitol for all three fluids with a significance level of *p* = 0.05 using the extra sum-of-squares F-test, if the overall F-score was significant.

### Pharmacokinetic analysis

Data for gentamicin concentration in blood were fit to a first-order, decreasing exponential because intravenous infusion resulted in maximum blood levels before the first sampling time at 0.5 h.

For gentamicin in blood the equation was:

$$ y(t)={y}_0{e}^{- kt}\kern13em eq.1 $$

Where y(t) is the concentration at time in hours t, y_0_ is the maximum concentration and k is the rate constant that represents the rate of change of the concentration. A quadratic fit to the data for perilymph and CSF determined that the peak concentration for perilymph and CSF occurred at about 4 h. For this reason the perilymph and CSF data were fit to a function that is the sum of an increasing exponential followed by a decreasing exponential with a peak at 4 h. For gentamicin in perilymph and CSF the equation was:

$$ y(t)={y}_{max}\left[{e}^{-{k}_1t}+\left(1-{e}^{-{k}_{2^{\left(t-4\right)}}}\right)\right]\kern6.5em eq.2 $$

Where k_1_ and k_2_ parameters are constants for concentration decrease and increase respectively, which determine the overall rate of change of the concentration for gentamicin. Larger “k” values indicate more rapid rate of change. Significant differences in permeability of the BLB due to mannitol are indicated if y_max_, k, k_1_ or k_2_ differ significantly between the with and without mannitol conditions. Another way to describe the rate of change of concentration that some readers may understand is the half-life (t_1/2_) which is the time required for the concentration to decrease by one-half. For a system with one decay constant, k_x_, the half-life (t_1/2_) can be calculated as ln(2)/k_x_ or t_1/2_ = 0.693/k_x_.

## Results

Data were adequate for convergence for all parameters in this study. Goodness-of-fit estimated by the *R* ^2^ statistic were considered large or larger than typical [18].

### Blood

**Fig. 1:**
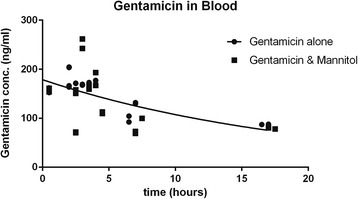
Gentamicin Concentration (ng/ml) over time in blood. Gentamicin was delivered intravenously so the maximum blood concentration was achieved before the first sample could be taken at about 0.5 h. Data were fit to a first-order, decreasing exponential as eq. 1: [*y*(*t*) = *y*_0_ *e* ^−*kt*^], to determine pharmacokinetic parameters rate constant k, and concentration at time t, when *t* = 0, y_0_. The model parameters were not statistically significantly different between the mannitol with and without conditions so the fit line shown is for the data without mannitol. The fit line is described by the function *y*(*t*) = 184*e* ^−0.045*t*^

The pharmacokinetics models for gentamicin in blood were not statistically significantly different between the model without gentamicin versus the gentamicin with mannitol model (Table 1, *p* = 0.433; *R* ^2^ = 0.61). This finding indicated that mannitol did not significantly change clearance from blood. The t_1/2_ of gentamicin in blood was 13.6 h, and the maximum concentration was 184 ng/ml. These results are graphically displayed in Fig. 1.

**Table 1:** Pharmacokinetic Parameters of Gentamicin Concentrations in Blood

Parameter	Gentamicin (ng/ml) given alone in blood (95%CI)	Gentamicin (ng/ml) with mannitol in blood (95%CI)	*p = 0.433*
Y_max_	184 (167–200)	172 (132–213)	
k	0.045 (0.025–0.066)	0.055 (0.002–0.109)	
Half life	15.27 (10.5–28.05)	12.50 (6.4–324)	
tau	22.03 (15.1–40.5)	18.03 (9.2–467)	
*R* ^2^	0.61	0.26	

**Fig. 2:**
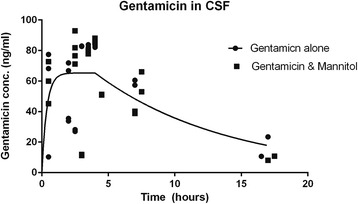
Gentamicin concentration (ng/ml) in Cerebrospinal Fluid (CSF). Data were fit to the sum of two first-order exponential equations and meant to model the sum of absorption and elimination of gentamicin from CSF. One equation was an increasing exponential starting at time t when t = 0 and the second a decreasing exponential starting when *t* = 4 h. A quadratic fit to the data suggested that the peak occurred when time was 4 hours. The overall equation is eq. 2, [$y(t)={y}_{max}\left[{e}^{-{k}_1t}+\left(1-{e}^{-{k}_{2^{\left(t-4\right)}}}\right)\right]$ where k_1_ and k_2_ parameters are rate constants for decrease and increase concentration (hours^−1^), respectively, which determine the overall rate of change of the concentration for gentamicin. The models with (*n* = 20) or without mannitol (*n* = 21) were not significantly different (*p* = 0.58) so the data for gentamicin concentration without mannitol are shown. As in Figs. 1 and 3 many data points are congruent so they look like one datum

**Fig. 3:**
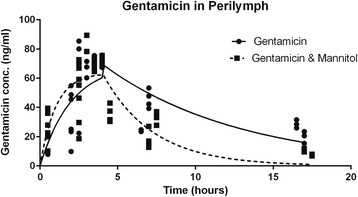
Gentamicin concentration (ng/ml) in perilymph. Data were fitted to the eq. 2 as in Fig. 2 but there were significant differences between the model with gentamicin (*n* = 43) and without gentamicin (*n* = 44) (*p* = 0.0044), indicating that mannitol changed the permeability of the blood-labyrinth barrier. As in Figs. 1 and 2, many data points are congruent so they look like one datum. The parameterized equations for gentamicin concentration without mannitol, then was [$y(t)=70\left[{e}^{-0.5t}+\left(1-{e}^{-{0.11}_{\left(t-4\right)}}\right)\right]$, and the equation with mannitol was $\Big[y(t)=64\left[{e}^{-t}+\left(1-{e}^{-{0.31}_{\left(t-4\right)}}\right)\right]$

Data were fit to a first-order, decreasing exponential as eq. 1: [*y*(*t*) = *y*_0_ *e* ^−*kt*^], to determine pharmacokinetic parameters rate constant k, and concentration at time t when *t* = 0 h, y_0_. Fitted parameters did not differ significantly (p = 0.433) with or without mannitol so the best fit gentamicin alone is shown (*n* = 22 with mannitol and n = 22 without mannitol). As in Figs. 2 and 3, many data points are congruent so they look like one datum. The fitted parameters for eq. 1, (for +/− 95% confidence interval), were y_0_ = 184 (+/− 167–200) ng/ml; k = 0.045(+/− 0.025–0.066) hrs^−1^. Parameterized eq. 1 is therefore: *y*(*t*) = 184*e* ^−0.045*t*^. An alternative way of expressing the rate of decay is the half-life t_1/2_, which was 15.3 (+/− 10.5–28.05) hours. R^2^ for these data was 0.61 indicating that about 61% of the variability is accounted for by the model. The finding that parameters k, and y_0_ were not different with or without mannitol indicate that mannitol did not significantly affect clearance of gentamicin from blood

### CSF

Figure 2 shows the “best-fit” pharmacokinetic parameters calculated by GRAPHPAD5 fit to eq. 2. The function was the sum of two exponentials. As for blood, the parameters of the models with and without mannitol were not statistically significantly different (Table 2, *p* = 0.58). The rate constant, k_1_ was 0.65 which is much larger than the rate constant for blood, k = 0.045 from Fig. 1, indicating that clearance is much more rapid from CSF than blood. As expected, the maximum concentrations (184 ng/ml in blood and 79 ng/ml in CSF) were much lower in CSF as well. Although clearance differs between blood and CSF, mannitol did not influence the clearance of gentamicin from blood or CSF significantly.

**Table 2:** Pharmacokinetic Parameters of Gentamicin Concentrations in CSF

Parameter	Gentamicin(ng/ml) given alone in CSF(95%CI)	Gentamicin(ng/ml) with mannitol in CSF(95%CI)	*p = 0.58*
Y_max_	79 (50–100)	66 (52–80)	
k_1_	0.65 (−0.25–1.3)	4.5 (−4.3–13)	
k_2_	0.11 (−0.026–0.25)	0.12 (−0.014–0.24)	
*R* ^2^	0.32	0.36	

Data were fit to the sum of two first-order exponential equations and meant to model the sum of absorption and elimination of gentamicin from CSF. The overall equation is eq. 2, [$y(t)={y}_{max}\left[{e}^{-{k}_1t}+\left(1-{e}^{-{k}_{2^{\left(t-4\right)}}}\right)\right]$ where k_1_ and k_2_ parameters are rate constants for decrease and increase concentration (hours^−1^), respectively, which determine the overall rate of change of the concentration for gentamicin. Y_max_ is the maximum gentamicin concentration (ng/ml) and y(t) is the concentration of gentamicin at time t (hours). Fitted parameters for eq. 2, (for +/− 95% confidence interval), were y_max_ = 79 (+/− 50–100) ng/ml; k_1_ = 0.65 (+/− −0.25-1.3) hrs^−1^; k_2_ = 0.11(+/− −0.026-0.25) hrs^−1^. Parameterized eq. 2 is therefore: [*y*(*t*) = 79[*e* ^−0.65*t*^ + (1 − *e* ^−0.11(*t* − 4)^)]. The half lives for decrease and increase were 1.07 and 6.3 h respectively. R^2^ was 0.32, indicating that 32% of the variability was accounted for by the model. This amount seems somewhat low, probably due to the difficulty in fitting the phase of increasing concentration. These data suggest that mannitol does not significantly change the permeability of the blood-brain barrier to gentamicin

### Perilymph

Unlike blood and CSF, the effect of mannitol on the permeability of the BLB to gentamicin was significant (Table 3, *p* = 0.0044). The *R* ^2^ values for the models for gentamicin concentration without and with mannitol were quite good, indicating that about 60% of the variability in the data was explained by the model (*R* ^2^ = 0.59 and 0.69 respectively). Data were fitted to eq. 2. Figure 3 illustrates the differences in permeability due to mannitol. Calculating t_1/2_ from k_1_ as for CSF, the half-life of gentamicin in perilymph was reduced from twelve hours to less than one hour by the addition of mannitol. The rapid increase in elimination of gentamicin induced by mannitol was much more significant compared to our controls, although the maximum concentration (Y_max_) remained unchanged between two groups.

**Table 3:** Pharmacokinetic Parameters of Gentamicin Concentrations in Perilymph

Parameter	Gentamicin(ng/ml) given alone in Perilymph (95%CI)	Gentamicin(ng/ml) with Mannitol in Perilymph (95%CI)	*p = 0.0044*
Y_max_	70 (55–84)	64 (55–72)	n.s.
k_1_	0.50 (0.25–0.75)	1.0 (0.42–1.6)	*
k_2_	0.11 (0.49–0.18)	0.31 (0.17–0.45)	*
*R* ^2^	0.59	0.69	

Data were fitted to the eq. 2 as in Fig. 2 but there were significant differences between the model with gentamicin (*n* = 43) and without gentamicin (*n* = 44) (*p* = 0.0044), indicating that mannitol changed the permeability of the blood-labyrinth barrier. Without mannitol and (+/− 95% confidence interval), the parameters were y_max_ = 70 (+/− 55–84) ng/ml; k_1_ = 0.50 (+/−0.25–0.75) hrs^−1^ with t_1/2_ = 12.6 h; k_2_ = 0.11(+/− 0.49–0.18) hrs^−1^ with (t_1/2_ = 6.3 h); R^2^ = 0.59. With mannitol and (+/− 95% confidence interval), the parameters were y_max_ = 64 (+/− 55–72) ng/ml; k_1_ = 1.0 (+/− 0.42–1.6) hrs^−1^ with t_1/2_ = 0.69 h; k_2_ = 0.31 (+/− 0.17–0.45) hrs^−1^ with t_1/2_ = 2.2 h; R^2^ = 0.69. Y_max_ is not statistically significantly different but k1 and k2 are (both *p* < 0.05), indicating that mannitol caused increased permeability of the blood-labyrinth barrier to gentamicin, allowing more rapid entry and egress and subsequently lower concentration. The parameterized equations for gentamicin concentration without mannitol, then was $\Big[y(t)=70\left[{e}^{-0.5t}+\left(1-{e}^{-{0.11}_{\left(t-4\right)}}\right)\right]$, and the equation with mannitol was $\Big[y(t)=64\left[{e}^{-t}+\left(1-{e}^{-{0.31}_{\left(t-4\right)}}\right)\right]$

Calculated sample size to demonstrate that the observed difference in gentamicin concentrations in perilymph with and without mannitol of 6 ng/ml was statistically significant at the *p* = 0.05 level was 59 samples or ears, ie 30 animals.

## Discussion

Our results suggest that mannitol increases the elimination of gentamicin from perilymph but not blood or CSF. The maximum concentrations of gentamicin in perilymph and CSF are similar (about 70–79 ng/ml for the dose and conditions in this study) and these findings suggest that the BLB and the BBB have similarities. In addition, biochemical studies have shown that for some substances, the composition, turnover, as well as protein concentration are considerably different between perilymph and CSF so perilymph must have a different origin [1, 19–21]. However, it appears that the BLB is more sensitive to mannitol than the BBB for gentamicin, similar to findings with salicylate [22]. This finding may permit manipulation of the levels of compounds in the cochlea. By controlling the times of administration of a drug compared to mannitol, drugs could either be eliminated more quickly or could achieve higher concentrations in perilymph before the action of mannitol abates. Our findings suggest that the BLB is not the same as the BBB and therefore perilymph is a unique fluid, and not identical to CSF.

The maximum gentamicin concentration in blood was 184 ng/ml as opposed to 70 ng/ml in perilymph and 79 ng/ml in CSF. The concentration of gentamicin in perilymph and CSF was always significantly lower than in blood confirming that there were existing barriers between blood and either perilymph or CSF.

Gentamicin levels in endolymph would be valid but, endolymph volume is less than 2 μl making is subject to measurement error and contamination with perilymph. Our findings have potential for therapeutic intervention but any application would require empiric evidence and pharmacokinetic modelling. For example, by controlling the relative administration time, compounds such as gentamicin could be removed more quickly from perilymph than CSF, reducing ototoxicity, but retaining the therapeutic effect for treatment for diseases such as meningitis. Administration of mannitol should reduce ototoxicity in gentamicin overdose.

Unlike most antibiotics, the killing of bacteria by aminoglycosides is dependent on the peak concentration of the aminoglycoside. For most antibiotics, bacterial killing, as well as the degree of ototoxicity, correlates with the cumulative dose and/or area-under-the-curve of the concentration versus time. In other words, if a massive dose of gentamicin were delivered but quickly eliminated by mannitol injection, maximum killing of bacteria with minimal ototoxicity should result. These considerations may become particularly important in some gram-negative infections.

Aminoglycosides such as streptomycin have lost favour in treatment of tuberculosis because resistant organisms have narrowed the therapeutic window, making toxicity more likely. Multi-drug resistant tuberculosis is becoming an international problem so the use of aminoglycosides with otoprotection may become a viable treatment.

While dosages of mannitol and gentamicin are available in the literature we were unable to find any study in which gentamicin levels were measured simultaneously in perilymph, CSF, and blood. Laurell et al. (2000) studied gentamicin and radioactive mannitol and concluded that mannitol had no effect on the BLB because changes in hearing were not observed but actual concentrations of gentamicin were not measured [2]. Their study also used low doses of radioactive mannitol. In this study, we used 250 mg/kg of 20% mannitol which is clinically comparable to human dosages to reduce intracranial pressure (Lexi drugs database).

In our pilot project, we found that gentamicin in single IV doses of 300 mg/kg and 100 mg/kg, were acutely fatal to the guinea pig. This is in contrast to the previous literature which used these high dosages intraperitoneally [23, 24]. High intravenous doses of aminoglycosides can cause acute neuromuscular blockade and paralysis. The therapeutic dose of 4 mg/kg was chosen to provide enough sensitivity with the ELISA kit to measure gentamicin concentration in different sample fluids.

The literature offers some idea of when gentamicin samples should be taken to quantify the pharmacokinetics in the three fluids, but the data are inconsistent with half-life of gentamicin reported from 1 h to 8 h, in rat, guinea pig to chinchilla [2, 25–27]. Our data indicated that the maximum concentration of gentamicin after intravenous administration in CSF and perilymph occurs at about 4 h and the t_1/2_ is about 1 hour. In some situations, mannitol administration 4 h after gentamicin administration should permit maximum antimicrobial effect and minimal ototoxicity.

The study limitations are the lack of characterization of physiologic effect on different transport processes such as endolymphatic route, as well as morphologic and functional effect on organ of Corti and spiral ganglion neurons. This is mainly due to our terminal surgery to collect different specimens such as perilymph and CSF. Future directions will assess and compare changes in the permeability of BLB with respect to different mannitol concentrations, histological characterization of the sensory cells and afferent auditory neurons, localization of traceable gentamicin, and functional hearing outcome.

This is the first study that simultaneously evaluated gentamicin levels in perilymph, blood and cerebrospinal fluid with and without mannitol with therapeutic drug levels. Controlling the permeability of the BLB should lead to new therapeutic options for clinicians.

## Conclusions

Mannitol administration transiently increases the permeability of the BLB. Potential clinical benefits may accrue from selected timing of administration of mannitol augmenting the rate of entry and egress of compounds such as gentamicin into and out of perilymph.

## Abbreviations

BBB: Blood-brain barrier
BLB: Blood-labyrinth barrier
CSF: Cerebrospinal fluid
ELISA: Enzyme-linked immunoassay
Half life: t_1/2_ = ln(2)/k_x_ or t_1/2_ = 0.693/k_x_ (k_x_ is decay constant of a system)
k: Rate constants for association and decay which determine the rate of change of the concentration for drug in units of hours^−1^.
R^2^: Coefficient of determination
y(t): Concentration at time in hours (t)
y_0_: Concentration when time = 0 h for decreasing exponential in ng/ml
y_max_: Maximum concentration in ng/ml
